# Effect of an Integrative Mobile Health Intervention in Patients With Hypertension and Diabetes: Crossover Study

**DOI:** 10.2196/27192

**Published:** 2022-01-11

**Authors:** Sang Woo Oh, Kyoung-Kon Kim, Sung Soo Kim, Su Kyung Park, Sangshin Park

**Affiliations:** 1 Department of Family Medicine Dongguk University Ilsan Hospital Dongguk University College of Medicine Gyeonggi-do Republic of Korea; 2 Department of Family Medicine Gachon University Gil Medical Center Gachon University College of Medicine Incheon Republic of Korea; 3 Department of Family Medicine Chungnam National University Hospital Chungnam National University College of Medicine Daejeon Republic of Korea; 4 Graduate School of Urban Public Health & Department of Urban Big Data Convergence University of Seoul Seoul Republic of Korea

**Keywords:** diabetes mellitus type 2, obesity, hypertension, mHealth, mobile phone

## Abstract

**Background:**

Obesity, hypertension, and type 2 diabetes mellitus (T2DM) are worldwide epidemics that inflict burdens on both public health and health care costs. Self-management plays an important role in the proper management of these 3 chronic diseases, and in this context, mobile health (mHealth) can be a cost-effective self-management tool.

**Objective:**

The aim of this pilot study is to evaluate the effects of an integrative mHealth approach for obesity, hypertension, and T2DM on body fat, blood pressure, and blood glucose levels and demonstrate the clinical outcomes. The participants were patients aged 40 to 70 years who were treated for T2DM (hemoglobin A_1c_ [HbA_1c_] above 6.0%) without insulin or hypertension and obesity, controlled with pharmacotherapy.

**Methods:**

This pilot study was performed using a controlled, randomized, 3-month, 2-period crossover design. A total of 37 participants were recruited from 2 university hospitals in South Korea. Integrative mHealth comprised 4 parts: self-measuring home devices for monitoring blood glucose and blood pressure; 2 smartphone apps, where one gathered lifestyle data, giving them feedback with health information, and the other provided drug information and reminders of the medication schedule; unmanned kiosks for official measurement of blood pressure and body composition; and web-based access to participants’ health information.

**Results:**

Data from the 32 participants were analyzed. Their mean HbA_1c_ level was 7.5% (SD 0.8, ranging from 6.1% to 9.4%). Approximately 38% (12/32) of the participants had hypertension. BMIs of all participants except 1 were >23 kg/m^2^. The input rates of food intake and exercise to the smartphone app were very low (24.9% and 5.3%, respectively). On the contrary, the input rate of medicine intake was high (84.0%). Moreover, there was no significant difference in the input rate of taking medicine irrespective of whether the mHealth period was before or after the conventional treatment period (80.3% and 87.3%, respectively; *P*=.06). Among the 3 input functions of food intake, exercise, and medicine intake in smartphone apps, the input of medicine intake was a more helpful, easier to use, and better-designed function than the others. There were no significant differences in changes in body weight (−0.519 kg vs 0 kg), BMI (−0.133 kg/m^2^ vs −0.167 kg/m^2^), body composition (body fat −0.255% vs 0.172%), blood pressure (systolic −0.226 mm Hg vs −2.839 mm Hg), and HbA_1c_ (−0.269% vs –0.009%) between the integrative mHealth and conventional treatment groups. However, in proportion to the elevation in the input rate of taking medicine, body fat mass (*P*=.04) and HbA_1c_ (*P*=.03) were lower in the integrative mHealth group.

**Conclusions:**

Although smartphone apps can influence body fat and blood glucose levels, they have failed to show clinical improvement. A higher input rate of taking medicine was related to significantly lower body fat mass and HbA_1c_ levels.

## Introduction

### Background

Obesity is the established main cause of hypertension and type 2 diabetes mellitus (T2DM) and can lead to the development of coronary vascular disease; furthermore, these 3 illnesses have attained the status of global epidemics recently [1-3]. Consequently, they inflict a huge economic burden on public health systems worldwide. Obesity was estimated to account for 0.7% to 2.8% of the total health care expenditure (HCE), and people with obesity had health care costs that were approximately 30% greater than those with normal weight [4]. The incremental medical expenditure ratios for people in Korea with BMIs of 30 kg/m^2^ to 34.99 kg/m^2^ and >35 kg/m^2^ were 34.3% and 38.4%, respectively, as compared that of with people with BMIs of 18.5 kg/m^2^ to 22.99 kg/m^2^ from 2002 to 2013 [5]. The US national health care spending associated with hypertension was estimated to be approximately US $131 billion, averaged over 12 years from 2003 to 2014 [6]. In 2017, the total estimated cost of diagnosed diabetes in the United States was US $327 billion, including medical costs and lost productivity, and provision of care for people with diagnosed diabetes accounted for a quarter of health care costs [7]. Without appropriate management of obesity, hypertension, and T2DM, patients experience disastrous complications, and societies are troubled with HCEs and disease-related productivity losses.

Self-management is crucial for the proper management of the 3 chronic diseases. A structured lifestyle intervention program comprising a healthy diet, physical activity, and behavioral interventions is essential for the treatment of obesity [8]. Lifestyle management and self-management with self-monitoring are also important in the treatment of hypertension and T2DM [9-11]. However, in face-to-face outpatient consultations, health care providers lack time to deliver information and skills for self-management to patients and motivate them to change their lifestyles.

In this context, mobile health (mHealth) can be a cost-effective tool for self-management in the treatment of chronic diseases. The Global Observatory for eHealth defined mHealth as a medical and public health practice supported by mobile devices [12]. It is useful as it can (1) enhance drug adherence through reminders, (2) facilitate self-monitoring coupled with wireless medical peripheral devices, and (3) provide tailored practical information.

A considerable number of clinical trials have been executed to inspect the usefulness of mHealth interventions in the treatment of obesity, hypertension, and T2DM [13,14]. The reviews and meta-analyses of these trials indicate that although mHealth interventions are likely to promote weight loss, lower hemoglobin A1c (HbA_1c_), and reduce blood pressure, the individual results are mixed [13,14]. In Korea, several groups have reported the clinical usefulness of smartphone-based apps in T2DM patients; however, their glucose-lowering effect is not clear [15-17]. Although numerous trials have investigated the efficacy of mHealth in the management of obesity, hypertension, and T2DM, they have explored its effect with respect to the 3 diseases, separately or in patients with obesity and hypertension or in patients with hypertension and T2DM. As hypertension and T2DM are comorbidities of obesity, and the 3 diseases are important risk factors of coronary vascular disease, integrative lifestyle approaches for the 3 diseases are more appropriate. They should include the feedback system of diet and exercise, medication assistance, and self-monitoring of blood pressure, blood glucose, and body composition.

### Objectives

The aim of this 6-month crossover pilot study is to evaluate the clinical effects of integrative mHealth supported by self-monitoring home devices among patients with T2DM or hypertension and obesity. The integrative mHealth used in this pilot study provides a platform to link the out-of-hospital self-monitoring results of diet, exercise, blood pressure, blood glucose, and body composition with web servers for data storage and web portals for the patient and their physician’s data access. Embedded apps in patients’ smartphones are LIBIT (Huraypositive Co) for recording diet and exercise, connecting self-measuring home devices to web servers, and providing feedback and health information to patients; and Mediram (GST Korea) for medication assistance. These smartphone apps have been newly developed for this project.

## Methods

### Study Participants

The pilot study was conducted with adults aged 40 to 70 years who were treated for T2DM (without insulin) or hypertension in the departments of family medicine and endocrinology at 2 university hospitals and were in stable status for at least the past 4 months. Recent HbA_1c_ levels of participants measured in <4 months were >6.0%. To use the Bluetooth-enabled self-measuring home devices and smartphone apps developed for this pilot study, the participants should have had and been able to use Android smartphones with OS version 4.3 (jellybean) or later. Recruitment was conducted between October 2018 and February 2020 in Incheon and Daejeon, which are 2 metropolitan cities in South Korea. The exclusion criteria comprised a history of malignant diseases, coronary artery obstructive disease, stroke, organ transplantation, drug abuse and alcohol dependence, disability or respiratory disease limiting exercise, and hospitalization in the past 6 months with major medical conditions.

All participants were informed of the aim and process of the pilot study during the interviews and were requested for their consent to join this study. The study procedures were performed only with participants who provided informed consent. This pilot study was approved by the institutional review boards of Gachon University Gil Medical Center and Chungnam National University Hospital, and the pilot study was performed in accordance with the Declaration of Helsinki and the guidelines of Good Clinical Practice. Deidentified and anonymized data were used in the analyses.

### Study Design, Devices, and Smartphone Apps

**Figure 1:**
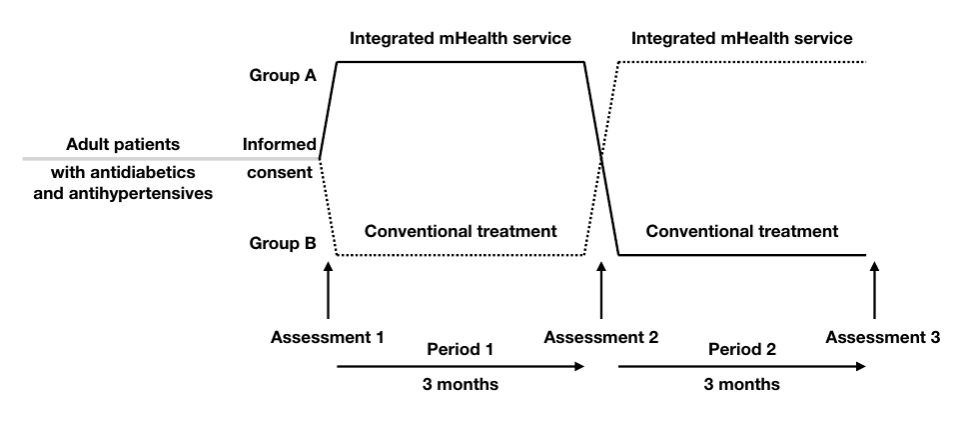
Study design. mHealth: mobile health.

This pilot study was performed using a controlled, randomized, 3-month, 2-period crossover design to test the efficacy of integrative mHealth coupled with self-monitoring home devices and smartphone apps as opposed to the conventional treatment (CON) of T2DM with or without hypertension. Figure 1 demonstrates the pilot study design. The recruited participants were randomly assigned to 2 groups using computer-generated random numbers: 1 group started with the integrative mHealth service period and switched to the CON period (mHealth-CON group), whereas the other group started with the CON period and switched to the mHealth period (CON-mHealth group). There was no washout period. Measurements of body weight, body composition, blood pressure, and HbA_1c_ were taken at the start of the first treatment period, during treatment transition, and at the end of the second treatment period. A survey on smartphone apps was conducted at the end of the pilot study.

**Figure 2:**
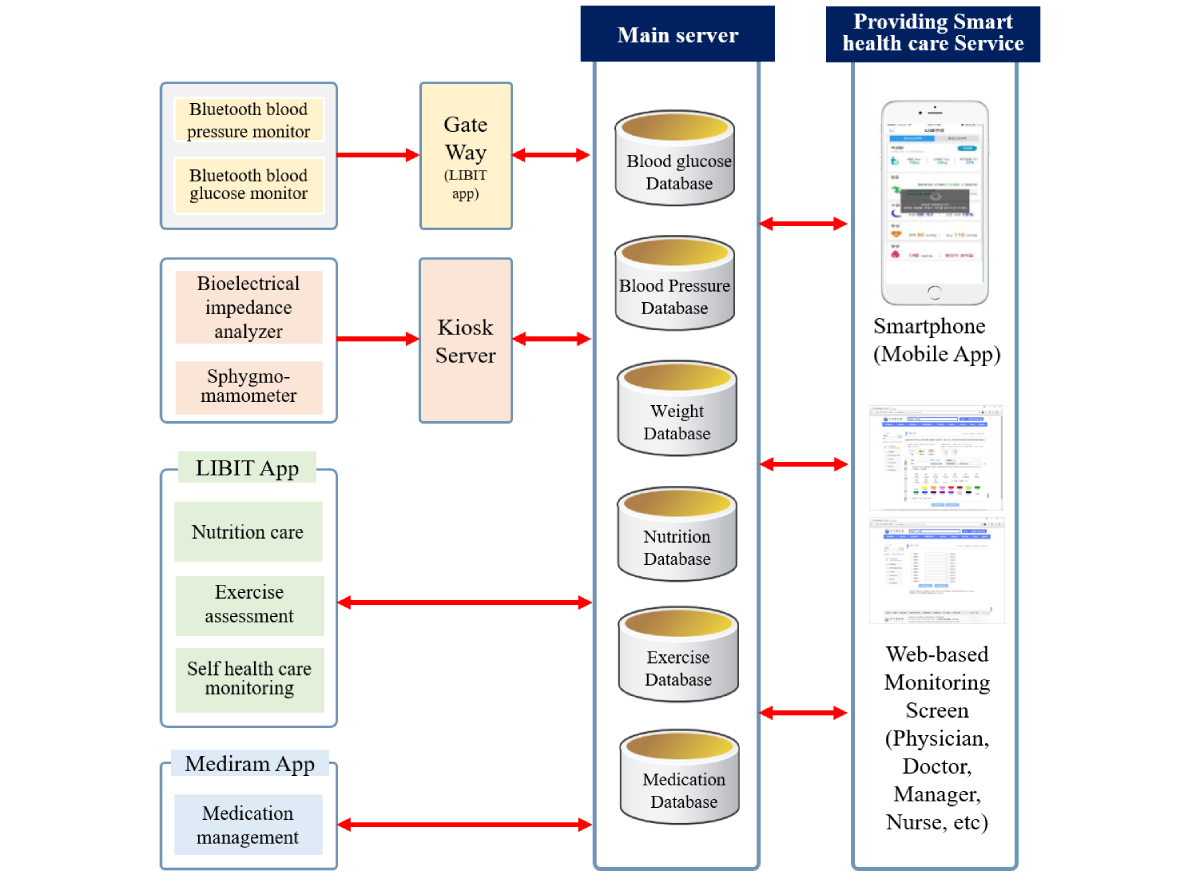
Architecture of information transmission.

Integrative mHealth supported the participants and physicians through the following 4 components: (1) self-measuring devices; (2) smartphone apps that gathered and transferred data on the participant’s lifestyle and provided feedback, health and drug information, and reminders of their medication schedule; (3) unmanned kiosks for the official measurement of blood pressure and body composition; and (4) web-based access to participants’ health information through which physicians could review participants’ health data at a glance. The entire architecture of the information transmission in this pilot study is shown in Figure 2. For systematic collection and administration of health information data, this pilot study emphasized data security by applying 5 systems: (1) section encoding via secure socket layer (SSL) or transport layer security, (2) encoding critical information, (3) controlling the users’ and administrators’ accessibility to data, (4) restricting the collection of personal identification information, and (5) agreeing to collect and use personal identification information. Information with a high risk of data loss, such as passwords, was saved using the unilateral encoding system of Secure Hash Algorithm 256 in the health information service system. Integrative mHealth was available only for the mHealth period. The devices and smartphone apps were supplied at the commencement of the mHealth period and retrieved at the end of it.

A total of 2 Bluetooth-enabled devices, the blood pressure monitor HEM-9200T (Omron) and the blood glucose monitor CareSense N Premier BLE (i-sense), were used as self-measuring home devices in this pilot study (Multimedia Appendix 1). A total of 2 unmanned digital kiosks (GST Korea) were established at both hospitals for the self-measurement of blood pressure and body composition (Multimedia Appendix 2). The kiosks were equipped with a user screen, a radio-frequency identification card reader, a bioelectric impedance analyzer SC-330 (Tanita), and an automatic upper arm sphygmomanometer BP-210 (Accuniq). For using the kiosks, a radio-frequency identification card was supplied to each participant.

The readings of blood pressure and glucose levels that were measured at home were transmitted to the patient’s Android smartphone app LIBIT through a Bluetooth connection and then transferred to the main server using cellular data. The data on body composition and blood pressure measured at the kiosks were also linked to the main server through http secure based on certificate verification; http secure sent encoded information of the clients to the server using the security protocol of SSL. SSL or transport layer security operates in the same way as a virtual private network, sending security data to the server via a virtual tunnel.

A total of 2 Android apps, LIBIT and Mediram, were developed to gather participants’ lifestyle data, provide feedback and health and drug information, and enhance their adherence to medication in this pilot study. The LIBIT app comprised 4 functions: nutrition care, exercise assessment, transmission of self-monitored blood pressure and glucose level, and health monitoring (Multimedia Appendix 3). The nutrition care component of LIBIT calculated the suggested total calorie and macronutrient ratios of each participant. Users could record their food intake through the smartphone keypad or their voice using embedded voice recognition technology. Analyzing the food intake records, LIBIT estimated the intake of 14 nutrients (total calories, carbohydrates, proteins, fats, calcium, phosphorus, iron, potassium, sodium, vitamin A, thiamine, riboflavin, niacin, and vitamin C) and reported each of them as insufficient, suitable, or excessive for the users. Users could also record the kind and duration of exercise through the exercise assessment function of LIBIT; it subsequently calculated the amount of calorie consumption and reported it to the users. LIBIT received participants’ self-monitored data on blood pressure and glucose from the peripheral devices through a Bluetooth connection and transmitted them to the main server using cellular data. The health monitoring function of LIBIT provided visual feedback of users’ health status by creating trend graphs of body weight, blood pressure, glucose level, and calorie consumption. The Mediram app offered comprehensive medication information to users (Multimedia Appendix 4). Users could easily upload the prescription to Mediram by just scanning the QR codes of their prescriptions. Mediram supplied drug information and notified users of their drug schedule to enhance their adherence to medication.

Physicians could access the participants’ health data using Bluetooth-enabled home devices and unmanned kiosks at the main server using web browsers (Multimedia Appendix 5). The average blood pressure, glucose level, adherence to medication, body composition change, nutrient intake, and calorie consumption through exercise in the given period were displayed on a page, and the physician could monitor changes in a participant’s health status at a glance.

### Statistical Analyses

The input rate of food intake was calculated as the total input frequency divided by the product of the days of the mHealth period and frequency of daily food intake. The input rate of taking medicine was calculated similarly as the total input frequency divided by the product of days of the mHealth period and frequency of daily drug intake. The input rate of exercise was calculated as the total input frequency divided by the number of days of the mHealth period.

Student *t* test, chi-square test, and Fisher exact test were used to determine the differences in baseline characteristics between the mHealth-CON and CON-mHealth groups. The Wilcoxon signed-rank test was conducted to test the difference in the input rates of diet, exercise, and medicine intake between the groups. A paired *t* test or Wilcoxon signed-rank test was performed to determine the differences in changes between the integrative mHealth and CON periods. Subsequently, according to the input rate of medicine intake, the estimated between-group differences in changes in the variables—obesity, hypertension, and T2DM—were calculated using generalized linear models, after adjusting for treatment (mHealth or CON) in model 1; treatment, group (mHealth-CON or CON-mHealth), and sex in model 2; and treatment, group, sex, and age in model 3. To compare the 3 input functions of the apps, a Kruskal–Wallis rank sum test [18] was conducted.

All statistical analyses were implemented in the R software version 4.0.3 (R Core Team), which is a language and environment for statistical computing. A 2-tailed *P*<.05 was considered statistically significant.

## Results

### Baseline Characteristics

A total of 37 participants were enrolled in this pilot study. Among the 37 participants, there were 3 (8%) cases of newly diagnosed T2DM, 1 (3%) case of bioelectrical impedance analysis showing error, and 1 (3%) case of dropout. In the final data set, 32 participants’ data were included, of whom 15 (47%) were allocated to the mHealth-CON group, whereas the remaining 17 (53%) were assigned to the CON-mHealth group.

Among the 32 participants, 23 (72%) were men. The mean age of participants was 56.8 years, ranging from 40 to 69 years. Of the 32 participants, 17 (53%) graduated from college or above. Approximately 38% (12/32) of participants had hypertension. Most participants were overweight and obese. According to the BMI classification of the Korean Society for the Study of Obesity [19], only 3% (1/32) of participants were in the normal range, that is, 20.8 kg/m^2^. Of the 32 participants, 7 (22%) were overweight, with BMIs ranging from 23 kg/m^2^ to 24.9 kg/m^2^; 20 (63%) had class 1 obesity, with a BMI range of 25 kg/m^2^ to 29.9 kg/m^2^; and BMIs of the remaining 4 (13%) participants were >30 kg/m^2^. The mean HbA_1c_ level was 7.5%, ranging from 6.1% to 9.4%. The baseline demographic and clinical characteristics of the 2 groups, mHealth-CON and CON-mHealth, were similar, except for the frequency of hypertension (Table 1). There were no significant differences in demographic characteristics, body weight and body composition, blood pressure, and HbA_1c_ levels between the groups.

**Table 1:** Participant baseline characteristics (N=32).

Group		mHealth^a^-CON^b^ (n=15)	CON-mHealth (n=17)	*P* value^c^	
**Sex, n (%)**					
	Male	9 (60)	14 (82)	.24	
Age (years), mean (SD)		58.9 (4.9)	55.1 (7.6)	.11	
**Education, n (%)**					.14
	Elementary school	1 (7)	1 (6)		
	Middle school	0 (0)	2 (12)		
	High school	8 (53)	3 (18)		
	College or above	6 (40)	11 (65)		
**Marital status, n (%)**					.99
	Divorced	1 (7)	1 (6)		
	Married	14 (93)	16 (94)		
**House, n (%)**					.40
	Apartment	9 (60)	10 (63)		
	Detached	2 (13)	5 (31)		
	Unit	2 (13)	1 (6)		
	Other	2 (13)	0 (0)		
Monthly income^d^, mean (SD)		5.3 (2.3)	5.6 (2.3)	.71	
Hypertension, mean (SD)		9 (60)	3 (18)	.01^e^	
Body weight (kg), mean (SD)		73.4 (9.4)	80.0 (11.5)	.09	
BMI (kg/m^2^), mean (SD)		26.8 (2.3)	27.8 (3.6)	.34	
Fat mass (kg), mean (SD)		22.3 (5.5)	22.8 (6.9)	.83	
Body fat (%), mean (SD)		30.5 (7.1)	28.4 (6.9)	.38	
Fat free mass (kg), mean (SD)		51.1 (9.0)	57.2 (9.5)	.07	
Body water (%), mean (SD)		51.8 (4.1)	53.0 (4.8)	.45	
**Blood pressure (mm Hg), mean (SD)**					
	Systolic	134.8 (11.4)	129.4 (13.2)	.23	
	Diastolic	81.5 (7.8)	78.8 (11.8)	.46	
HbA_1c_^f^ (%), mean (SD)		7.5 (0.7)	7.5 (0.8)	.98	

^a^mHealth: mobile health.

^b^CON: conventional treatment.

^c^Calculated using Fisher exact test or Student *t* test.

^d^1: none, 2: <1 million Korean won (KRW), 3: KRW 1-2 million, 4: KRW 2-3 million, 5: KRW 3-4 million, 6: KRW 4-5 million, 7: KRW 5-6 million, 8: > KRW 6 million.

^e^Calculated using chi-square test.

^f^HbA_1c_: hemoglobin A_1c_.

### Diet and Exercise

The input rates of food intake (24.9%) and exercise (5.3%) were very low (Table 2). Moreover, both input rates were significantly low in the CON-mHealth group, which means that there was attrition in food intake and exercise input to the LIBIT app over time during the pilot study. On account of low input rates of food intake and exercise, it was impossible to execute the analysis of data related to nutrient intake and energy consumption.

**Table 2:** Input rates of diet, exercise, and taking medicine (N=32).

Parameters	mHealth^a^-CON^b^	CON-mHealth	Total	*P* value^c^
Diet, mean (SD)	36.6 (39.3)	14.5 (23.7)	24.9 (33.3)	.03
Exercise, mean (SD)	8.0 (9.3)	2.9 (8.9)	5.3 (9.3)	.002
Taking medicine, mean (SD)	80.3 (20.0)	87.3 (20.8)	84.0 (20.4)	.06

^a^mHealth: mobile health.

^b^CON: conventional treatment.

^c^Calculated using Wilcoxon rank sum test.

### Drug Adherence

The input rate of medicine intake to the Mediram app was high, at 84.0%. Unlike the input rates of food intake and exercise, there was no attrition in the input of medicine intake. There was no significant difference in the input rate of taking medicine irrespective of whether the mHealth period was before or after the CON period (80.3% and 87.3%, respectively; *P*=.06; Table 2).

#### Efficacy of mHealth on Body Weight, Body Composition, Blood Pressure, and HbA_1c_

The changes in body weight and body composition, blood pressure, and HbA_1c_ between the mHealth and conventional periods are displayed in Table 3. There were no significant differences in the changes in the variables of obesity, hypertension, and T2DM. The individual changes in these variables are shown in Multimedia Appendix 6.

**Table 3:** Comparison of the changes in body weight, body composition, blood pressure, and HbA_1c_^a^.

Characteristics		N (mHealth^b^/CON^c^)	mHealth, mean (SD)	Conventional, mean (SD)	*P* value^d^
Body weight (kg)		32/32	–0.519 (1.655)	0.000 (1.832)	.29
BMI (kg/m^2^)		32/32	–0.133 (0.640)	–0.167 (0.709)	.86
Fat mass (kg)		31/30	–0.292 (1.964)	0.153 (2.357)	.56^e^
Body fat (%)		31/30	–0.255 (2.454)	0.172 (2.574)	.43^e^
Fat free mass (kg)		31/30	–0.102 (1.703)	–0.173 (1.933)	.78^e^
Body water (%)		30/30	0.240 (3.173)	–0.360 (2.702)	.21
**Blood pressure (mm Hg)**					
	Systolic	31/31	–0.226 (12.328)	–2.839 (11.097)	.51
	Diastolic	31/31	–2.839 (9.000)	–1.097 (9.239)	.51^e^
HbA_1c_^f^ (%)		32/32	–0.269 (0.663)	–0.009 (0.693)	.19

^a^Some participants’ data are missing.

^b^mHealth: integrative mobile health service.

^c^CON: conventional treatment.

^d^Calculated by paired *t* test.

^e^Calculated using Wilcoxon signed-rank test.

^f^HbA_1c_: hemoglobin A_1c_.

#### Effect of the Medication Assistance App on Body Weight, Body Composition, Blood Pressure, and HbA_1c_

To inspect the effect of mHealth apps on body weight and composition, blood pressure, and HbA_1c_, the estimated changes in variables, according to the input rate of medicine intake, were calculated using generalized linear models (Table 4). *Group* was included as a variable in models 2 and 3 as there were differences in the prevalence of hypertension between the groups mHealth-CON and CON-mHealth. In proportion to the elevation of the input rate of medicine intake, body fat mass and HbA_1c_ were lower (Table 4). Owing to low input rates, the effects of food intake and exercise-related app functions on changes in the variables could not be assessed.

**Table 4:** The estimated changes in variables—obesity, hypertension, and type 2 diabetes mellitus—according to the input rate of taking medicine.

Characteristics		Model 1^a^			Model 2^b^			Model 3^c^	
		Estimate	*P* value	Estimate		*P* value	Estimate		*P* value
Body weight (kg)		–0.016	.16	–0.019		.09	–0.019		.11
BMI (kg/m^2^)		–0.004	.34	–0.005		.25	–0.005		.31
Fat mass (kg)		–0.027	.08	–0.034		.03	–0.032		.04
Body fat (%)		–0.030	.10	–0.037		.05	–0.035		.07
Fat free mass (kg)		0.007	.61	0.010		.49	0.008		.55
Body water (%)		0.035	.11	0.042		.07	0.040		.08
**Blood pressure (mm Hg)**									
	Systolic	–0.006	.95	–0.016		.85	–0.014		.87
	Diastolic	–0.016	.80	–0.028		.67	–0.028		.68
HbA_1c_^d^ (%)		–0.008	.07	–0.009		.04	–0.010		.03

^a^Model 1 adjusted for treatment (mobile health [mHealth] or conventional treatment [CON]).

^b^Model 2 adjusted for treatment, group (mHealth-CON or CON-mHealth), and sex.

^c^Model 3 adjusted for treatment, group, sex, and age.

^d^HbA_1c_: hemoglobin A_1c_.

### Survey on the Smartphone Apps

The results of the smartphone app survey at the end of the pilot study are summarized in Tables 5 and 6. Among the 3 input functions of food intake, exercise, and medicine intake in smartphone apps, the input of medicine intake was a more helpful, easier to use, and better-designed function than the others. There were more opinions about improvements in the input of food intake. The 2 most difficult functions were those of recording food intake and finding food items in the provided list. For the input of exercise, multitasking with other apps was highly desired.

**Table 5:** Survey about the functions of smartphone apps for the input of food intake, exercise, and taking medicine (Likert scale result; N=32).

Function of the apps and question		Likert scale (1-5), mean (SD)
**Input of food intake**		
	Helpful^a^	3.4 (1.0)
	Easy^b^	2.9 (1.2)
	Well-functioned^c^	3.1 (0.9)
**Input of exercise**		
	Helpful^a^	3.6 (1.0)
	Easy^b^	3.3 (1.0)
	Well-functioned^c^	3.3 (0.9)
**Input of taking medicine**		
	Helpful^a^	4.1 (0.8)
	Easy^b^	4.1 (0.7)
	Well-functioned^c^	4.0 (0.9)

^a^*P=*.01 using the Kruskal-Wallis rank sum test.

^b^*P<*.001 using the Kruskal-Wallis rank sum test.

^c^*P=*.002 using the Kruskal-Wallis rank sum test.

**Table 6:** Survey about the functions of smartphone apps for the input of food intake, exercise, and taking medicine (opinions; N=32).

Opinions			Numbers, n (%)	
**Input of food intake**				
	Have experience of using the diet app		1 (3)	
	**What needs to be improved?**		12 (38)	
		Difficult to record food intake		4 (13)
		Errors in voice recognition		1 (3)
		Wish the portion size was broken down		2 (6)
		Wish to input data on my own		1 (3)
		No food in the food list		5 (16)
		Unbelievable calculated calorie		1 (3)
**Input of exercise**				
	**What needs to be improved?**		7 (22)	
		Wish it worked with other apps simultaneously		4 (13)
		Wish to input data on my own		1 (3)
		Wish it was recorded automatically		2 (6)
		Difficult to input data		1 (3)
**Input of taking medicine**				
	**What needs to be improved?**		5 (16)	
		Errors in running the app		1 (3)
		Not easy to use		2 (6)
		Wish to go back to home screen after the input		1 (3)
		Wish to control the medication time		1 (3)

## Discussion

### Principal Findings

This pilot study demonstrated that smartphone apps could influence changes in body fat and blood glucose levels. As the input rate of medicine intake increased, body fat mass and HbA_1c_ decreased. Although the improvement in drug adherence for diabetes is expected to enhance the control of blood glucose levels, good adherence to antidiabetes medication is irrelevant to body fat reduction; however, some—not all—antidiabetes medicines can induce body weight loss [20]. This relationship can be better explained by positive behavioral changes and effective self-management skills obtained from the integrative mHealth intervention. These findings imply that mHealth can improve body fat and blood glucose status in patients with T2DM or hypertension; however, it failed to result in clinical improvement in this pilot study. In addition, an app for drug information and reminders is more pleasing to the eyes of the patients than a diet diary and exercise monitor. A larger and long-term clinical trial is needed to determine whether integrative mHealth services help patients with T2DM or hypertension and obesity. The findings of this pilot study are currently being applied to an improved mHealth intervention project for people who have moved into a large, new apartment complex.

A special feature of this pilot study is its crossover design. This pilot study allocated participants to 2 different interventions over two 3-month periods. A crossover design has the following advantages over a parallel design: (1) it may offer more precise estimates of intervention effects as it would remove the differences in participants’ characteristics and methodological variations of open trials, and (2) it requires a smaller number of participants [21,22]. Obesity, hypertension, and T2DM are chronic diseases and are appropriate for a crossover study as the conditions of patients are stable if the prescriptions do not change, and they are not usually curable. In addition, a crossover design was appropriate as integrative mHealth service was an add-on treatment to the CON and not a separate stand-alone treatment.

Although there was a chance of carryover effect in this pilot study, it could not have widened the differences in changes between the treatment groups. A washout period was logically impossible for the transition from CON to the add-on integrative mHealth intervention. There might be a carryover effect in the mHealth-CON group, which could be the reason that integrative mHealth could not induce significant clinical improvement as compared with CON alone. According to previous studies [14,23], mHealth has a clinically positive effect on chronic disease management. In the case of the carryover effect, it would strengthen the positive effect of the second phase of the treatment, that is, CON; subsequently, the differences in the 2 treatment phases would become smaller, which would make the analysis more conservative.

The input rate of taking drugs was high, that is, >80%, even in the CON-mHealth group, where Mediram use was assigned in the second 3-month period. In fact, it was marginally higher in the CON-mHealth group than in the mHealth-CON group. Pharmacological adherence is very important for the successful management of hypertension and diabetes. Among adults with several common chronic diseases, only 40% to 70% of medications are taken properly [24,25]. Poor drug adherence might lead to poor clinical outcomes and increased HCE in chronic diseases [26]. The use of mHealth may increase medication adherence in chronic diseases and coronary heart disease; however, the results are diverse [27-29]. The variation among the previous trials was probably because of the different modules of mHealth that facilitated proper drug intake. It appears that the medication assistance app Mediram independently improved body fat and blood glucose status in our pilot study. Further research is needed to verify the clinical usefulness of the medication assistance app and the features of the app that make it more effective.

On the contrary, the nutrition and exercise care app LIBIT was not popular among most participants. Although LIBIT introduced a voice recognition technique for diet diaries to make food intake input much easier, the mean input rate of food intake was only 24.9%. The self-recording rate of exercise was even lower, at 5.3%. Following the low input rates of food intake and exercise, individualized lifestyle feedback based on the amount of macro- and micronutrient intake and calorie consumption through exercise was not properly reported to the participants. Most of all, for the individualized lifestyle feedback system, the input methods of diet and activity should be easy and simple, as revealed by the participants.

The integrative mHealth service of this pilot study failed to clinically reduce body weight and fat, lower blood pressure, and improve T2DM. The amount of HbA_1c_ reduction in the mHealth period in our pilot study (–0.269%) was smaller than that in previous reports. A total of 2 meta-analyses reported that mHealth interventions improved HbA_1c_ significantly, with mean differences of –0.39% and –0.44%, respectively [14,23]. However, for obesity and blood pressure, the results of previous studies were mixed, and there might be attrition of improvement over time. A randomized controlled trial for 3 months in patients with T2DM and hypertension reported that the blood pressure differences between the intervention and control groups were narrowed during the second and third months compared with that of the first month [30]. Wang et al [13] outlined that mHealth induced an average weight loss widely ranging from –1.97 kg in 16 weeks to –7.1 kg in 5 weeks and mentioned that most studies were executed with small samples and in short intervention periods and did not use proper data collection or analytical methods. A reason for the failure of our mHealth intervention in improving body fat, blood pressure, and blood glucose levels might be the unsuccessful individualized lifestyle modification linked with the neglected input of food intake and exercise, considering the high input rate of medicine intake linked with the decrease in body fat and HbA_1c_.

Diabetes and hypertension are 2 major chronic diseases that incur burdens on public health economically, and the burdens will be bigger in the near future of the aging world. Life expectancy (LE) has increased drastically over the past several decades. Between 1950 and 2017, it increased from 48.1 years to 70.5 years for men and from 52.9 years to 75.6 years for women worldwide [31]. However, there is a big gap between LE and health-adjusted LE (HALE). Although the global average HALE increased from 57.6 years in 1995 to 63.3 years in 2017, the gap between LE and HALE also increased from 8.6 years to 9.7 years during the same period [32]. Population aging has been considered a big source of increase in HCE, and individual health status has been suggested as a main factor of HCE in the aging population [33]. Older adults who are hypertensive patients are more likely to have complications, including congestive heart failure or chronic kidney disease, and these comorbidities induce incremental medical expenditures for adults aged ≥65 years, which is approximately US $2500 more than that for adults aged 18 to 44 years [6]. Similarly, the prevalence of diabetes and the medical costs related to diabetes are primarily increasing among the population aged ≥65 years [7]. Integrative mHealth can be a cost-effective tool to prevent catastrophic complications and increased HCE, which are associated with hypertension and diabetes management in the aging population, by enhancing their self-monitoring skills and drug adherence.

There were some limitations to our pilot study. First, the treatment period was short, at 3 months. Until now, the long-term efficacy of mHealth has been doubtful, and one cannot be sure if its effect would wear down over time. Long-term clinical trials with serial assessments of their effects are necessary for the future. Second, only a few participants were prompt in recording their input of food intake and exercise on the lifestyle app. Consequently, without the availability of individualized lifestyle information, the integrative mHealth service in this pilot study was scaled down to the combination of self-monitoring of blood pressure and glucose levels, medication assistance app, unmanned kiosks, and physicians’ access to participants’ health records through a web browser. In addition to simpler and easier input methods of diet and exercise, more immersive and highly functional mHealth apps should be designed that focus in depth on the content and user experience and can motivate patients. Third, bioelectrical impedance analysis was used for the measurement of body composition. For the assessment of treatment effects on body composition, dual-energy X-ray absorptiometry would be appropriate, as bioelectrical impedance analysis is easily affected by the hydration status of the body. Finally, this pilot study was conducted with relatively young adults and not with older adults. Only 6% (2/32) of participants were aged >65 years. As chronic disorders are more common among older adults who may not have good digital literacy and have difficulty in adopting new information technologies, the apps and peripheral medical devices that are designed for self-monitoring should be better accessible for older adults. In addition, voice-based mHealth may be preferred by older adults with limited digital literacy and poor vision [34].

### Conclusions

This pilot study illustrated that smartphone apps could influence changes in body fat and blood glucose; however, mHealth failed to result in clinical improvement. A higher input rate of medicine intake was related to a significantly lower body fat mass and HbA_1c_. This result could possibly be because of positive behavioral changes and effective self-management skills obtained from the integrative mHealth intervention. In addition, the app for drug information and reminders was considered to be more pleasing to the eyes of the patients than a diet diary and exercise monitor.

## Appendix

Multimedia Appendix 1 (a) Bluetooth blood pressure monitor and (b) Bluetooth blood glucose monitor.

Multimedia Appendix 2 Nonhuman kiosks comprising a bioelectrical impedance analyzer, a user screen, a mobile health service radio-frequency identification card reader, and an automatic upper arm blood pressure monitor.

Multimedia Appendix 3 Smartphone screen of the LIBIT app for (a) nutrition and (b) exercise assessment.

Multimedia Appendix 4 Smartphone screen of the Mediram app.

Multimedia Appendix 5 Participant’s health data access through web browser.

Multimedia Appendix 6 Line plots of individual changes in (a) body weight, (b) BMI, (c) body fat mass, (d) body fat percentage, (e) fat free mass, (f) body water percentage, (g) systolic blood pressure, (h) diastolic blood pressure, and (i) HbA_1c_. mHealth: integrative mobile health service; CON: conventional treatment.

## Abbreviations

CON: conventional treatment
HALE: healthy life expectancy
HbA_1c_: hemoglobin A_1c_
HCE: health care expenditure
LE: life expectancy
mHealth: mobile health
SSL: secure socket layer
T2DM: type 2 diabetes mellitus
