# Supplementary figures and images for: Effect of an Integrative Mobile Health Intervention in Patients With Hypertension and Diabetes: Crossover Study
Source: JMIR Mhealth Uhealth. 2022 Jan 11;10(1):e27192. doi: 10.2196/27192 (PMC8790692; doi:10.2196/27192)

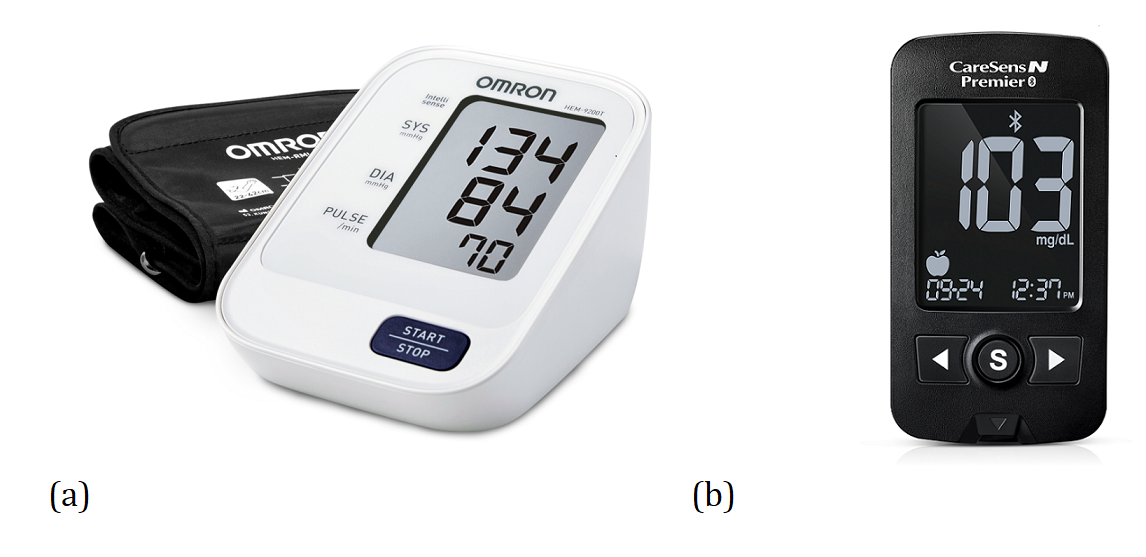

Supplement: Multimedia Appendix 1 [file mhealth_v10i1e27192_app1.png]

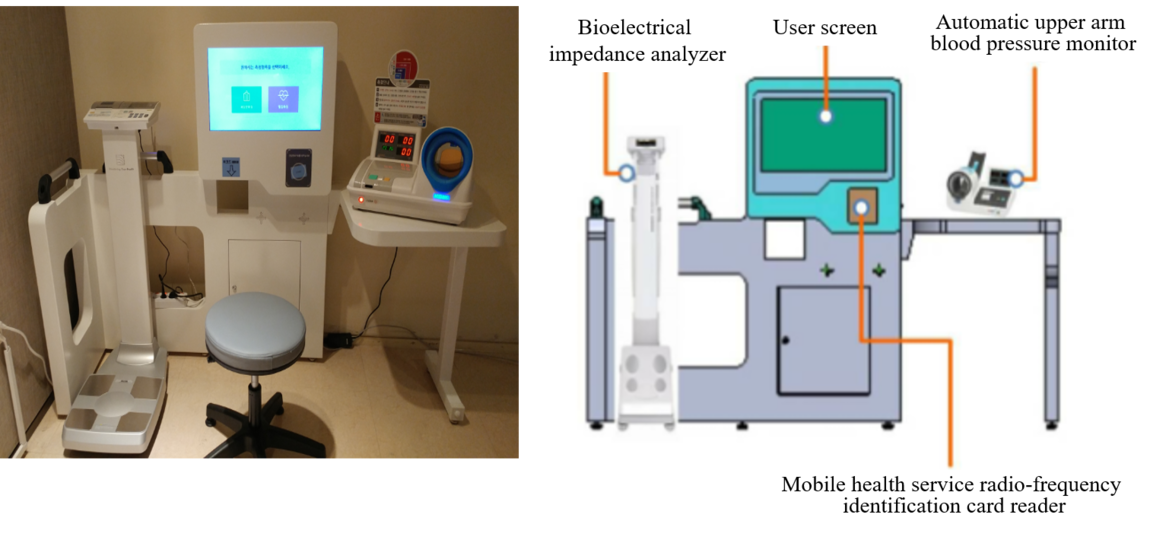

Supplement: Multimedia Appendix 2 [file mhealth_v10i1e27192_app2.png]

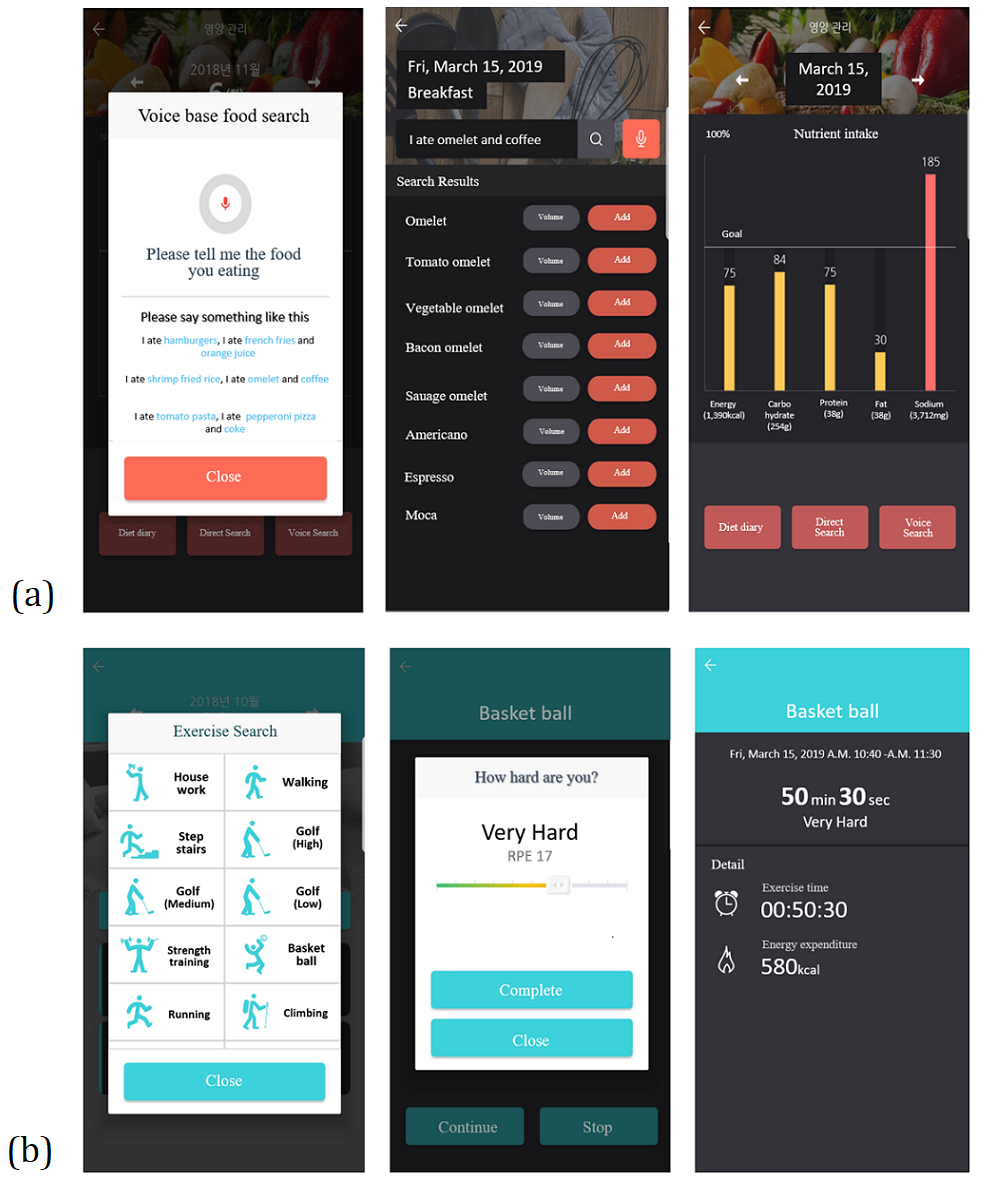

Supplement: Multimedia Appendix 3 [file mhealth_v10i1e27192_app3.png]

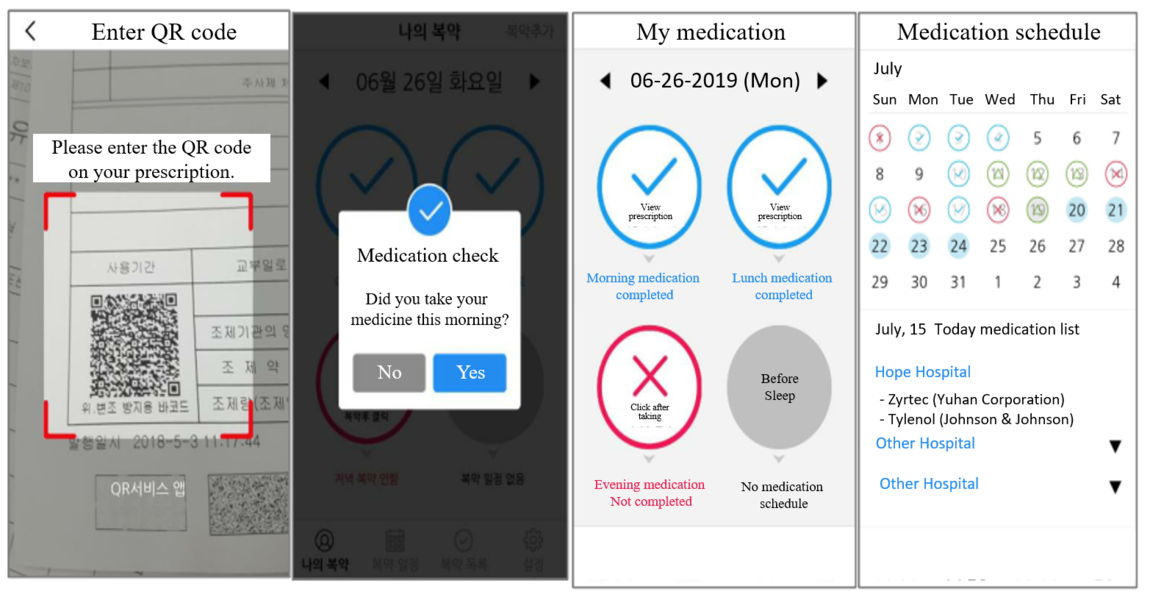

Supplement: Multimedia Appendix 4 [file mhealth_v10i1e27192_app4.png]

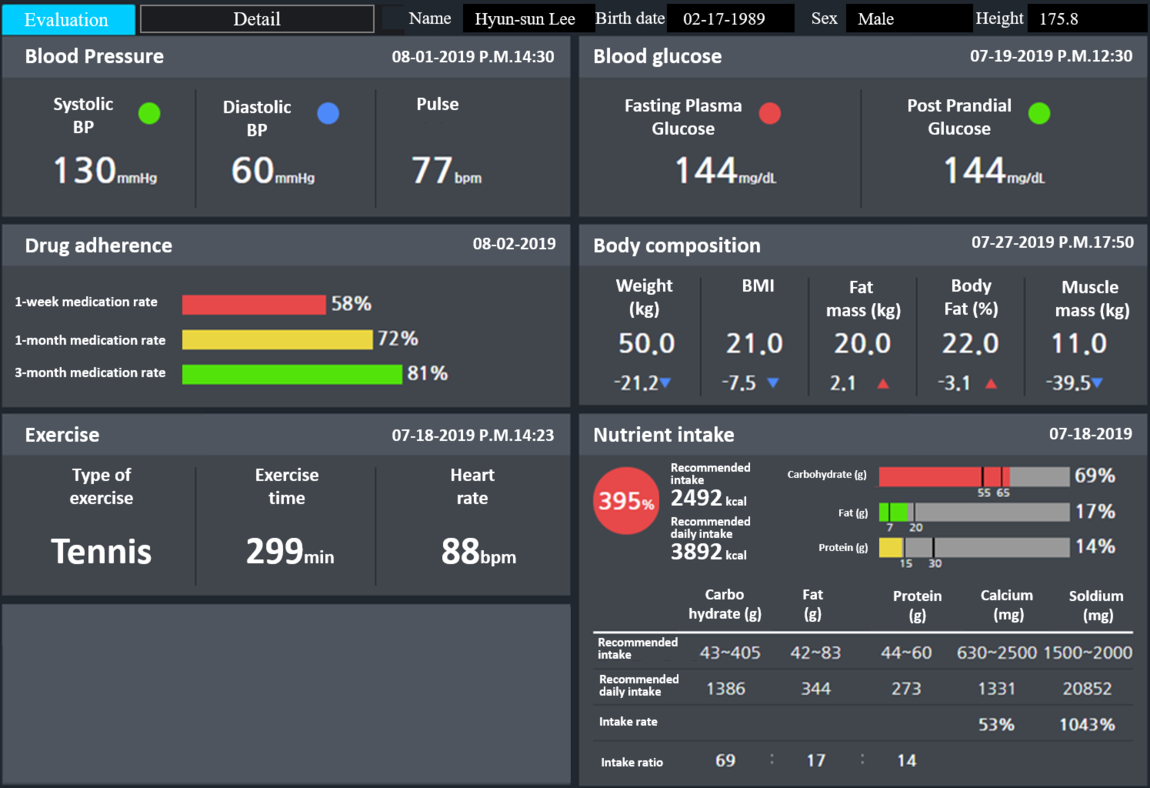

Supplement: Multimedia Appendix 5 [file mhealth_v10i1e27192_app5.png]

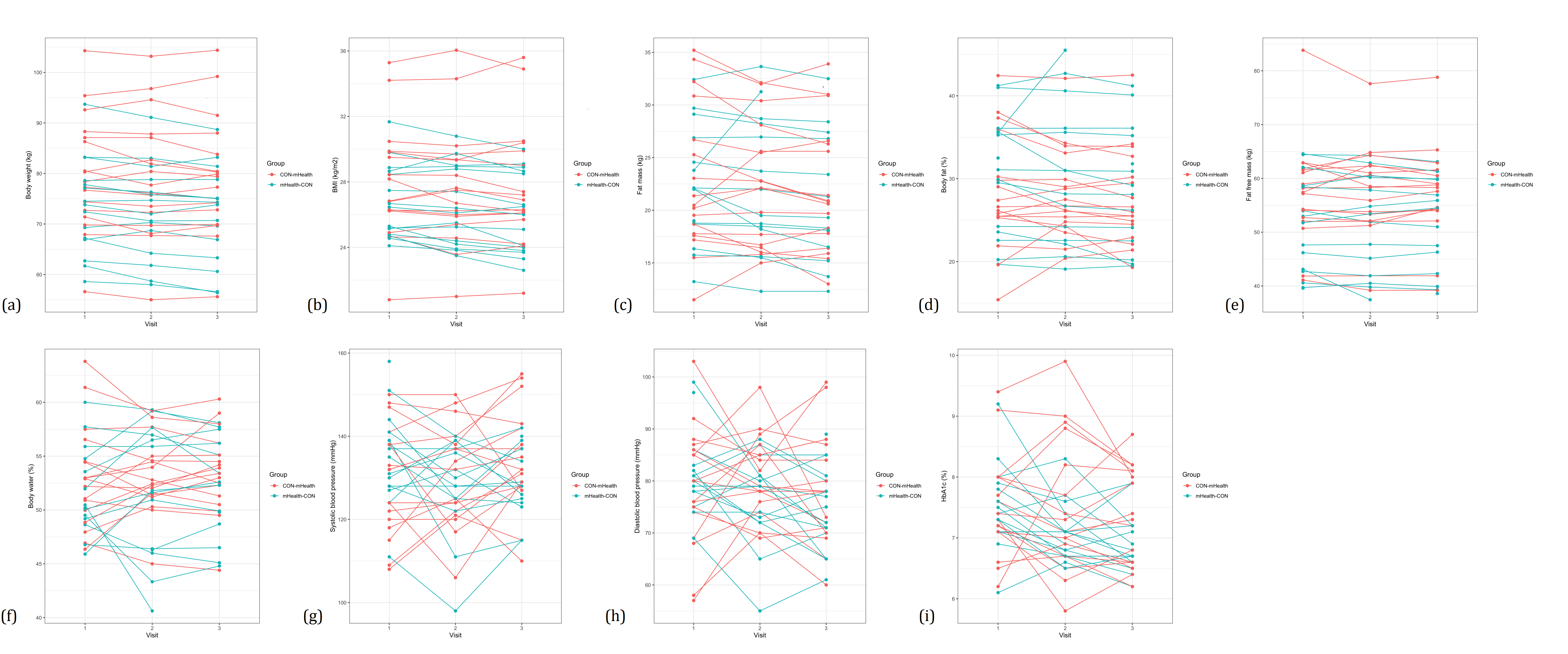

Supplement: Multimedia Appendix 6 [file mhealth_v10i1e27192_app6.png]
